# Central nervous system Langerhans cell histiocytosis and neurodegenerative syndrome responding to MEK inhibition

**DOI:** 10.1002/jha2.294

**Published:** 2021-10-30

**Authors:** Andrew Wahba, Branko Cuglievan

**Affiliations:** ^1^ Division of Pediatrics and Patient Care The University of Texas MD Anderson Cancer Center Houston Texas USA

A 13‐year‐old previously healthy girl presented with acutely worsened headaches, somnolence, memory lapses, ataxia, and hallucinations. Noted to have rib cage tenderness on physical examination and a chest X‐ray demonstrated lytic rib lesions, which were fluorodeoxyglucose (FDG)‐avid in positron emission tomography/computed tomography. Lesions in the ischium were also identified (Figure 1A). A bone biopsy was performed, and the histologic sections showed replacement of marrow by histiocytoid cells and scattered eosinophils with associated fibrosis (Figure 1B, H&E 200×). The cells were strongly positive for CD1a (Figure 1C, 200×) and S100 protein, characteristic for Langerhans cell histiocytosis (LCH). Next‐generation sequencing (NGS) molecular analysis demonstrated positivity for *BRAF* exon 12 deletion. *BRAFV600E*, *H3k27M*, and *IDH1* were negative. A magnetic resonance imaging (MRI) of the brain exhibited a T2 hyperintense enhancing mass arising from the optic chiasm/hypothalamus and extending into the floor of the third ventricle and into the interpeduncular cistern. The mass measured 2 × 1.8 × 1.9 cm. The T2/flair hyperintensity was classic for neurodegenerative syndrome (ND). She was started on vinblastine, corticosteroids, and cytarabine for three cycles, but she developed numerous endocrine complications. She was switched to cytarabine and trametinib and completed nine cycles. MRI brain with contrast demonstrates complete metabolic response and significant improvement in the size of the hyperintense enhancing mass arising from the hypothalamus (Figure 1D–F). She continues to be in remission with normal mentation and activity.

**FIGURE 1:**
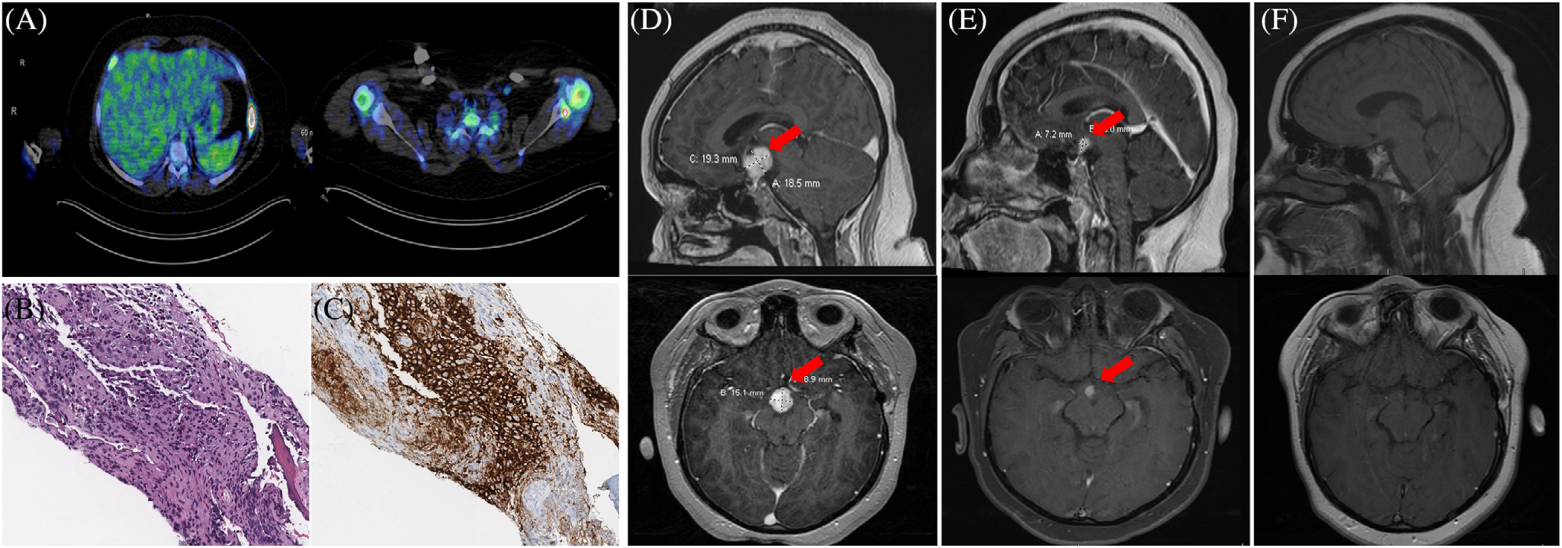
(A) Positron emission tomography (PET) scan showing lytic FDG‐avid lesions in the ischium and ribs. Tumor sections analyzed via hematoxylin and eosin (H&E) (B) and special stain (C) showing replacement of marrow by histiocytoid cells and scattered eosinophils with associated fibrosis (200× magnification) and cells were CD1a positive. (D–F) Magnetic resonance imaging (MRI) brain with contrast showing significant improvement in the size of the hyperintense enhancing mass arising from the hypothalamus

LCH is a rare myeloid neoplasia of CD1a+/CD207+ dendritic cells. There is no established optimal therapy for refractory CNS LCH or ND. *BRAF* exon 12 encodes the β3‐αC loop and is required for the kinase activation. The deletions in exon 12 render *BRAF* resistant to specific *BRAF‐V600* inhibitors like vemurafenib or dabrafenib. Since mutations in exon 12 activate the ERK/MAPK pathway, a MEK inhibitor was a reasonable targeting option and led to excellent results.

## CONFLICT OF INTEREST

The authors declare no conflict of interest.

